# Stress and Reproductive Hormones in Grizzly Bears Reflect Nutritional Benefits and Social Consequences of a Salmon Foraging Niche

**DOI:** 10.1371/journal.pone.0080537

**Published:** 2013-11-27

**Authors:** Heather M. Bryan, Chris T. Darimont, Paul C. Paquet, Katherine E. Wynne-Edwards, Judit E. G. Smits

**Affiliations:** 1 Faculty of Veterinary Medicine, University of Calgary, Calgary, Alberta, Canada; 2 Raincoast Conservation Foundation, Bella Bella, British Columbia, Canada; 3 Department of Geography, University of Victoria, Victoria, British Columbia, Canada; 4 Faculty of Environmental Design, University of Calgary, Calgary, Alberta, Canada; Federal University of Parana (UFPR)) – Campus Palotina, Brazil

## Abstract

Physiological indicators of social and nutritional stress can provide insight into the responses of species to changes in food availability. In coastal British Columbia, Canada, grizzly bears evolved with spawning salmon as an abundant but spatially and temporally constrained food source. Recent and dramatic declines in salmon might have negative consequences on bear health and ultimately fitness. To examine broadly the chronic endocrine effects of a salmon niche, we compared cortisol, progesterone, and testosterone levels in hair from salmon-eating bears from coastal BC (n = 75) with the levels in a reference population from interior BC lacking access to salmon (n = 42). As predicted, testosterone was higher in coastal bears of both sexes relative to interior bears, possibly reflecting higher social density on the coast mediated by salmon availability. We also investigated associations between the amount of salmon individual bears consumed (as measured by stable isotope analysis) and cortisol and testosterone in hair. Also as predicted, cortisol decreased with increasing dietary salmon and was higher after a year of low dietary salmon than after a year of high dietary salmon. These findings at two spatial scales suggest that coastal bears might experience nutritional or social stress in response to on-going salmon declines, providing novel insights into the effects of resource availability on fitness-related physiology.

## Introduction

Understanding the physiological responses of organisms to stressors is essential in predicting long-term consequences of environmental change [1]–[3]. Food limitation is a stressor that can directly affect population productivity by altering survival and reproduction [4]. Moreover, the distribution, abundance, and quality of food affect populations by mediating social structure and behavior [5]. Monitoring physiological indicators of nutritional and social stress may provide an early warning of population-level responses to environmental change [6]; this approach is particularly valuable in taxa such as ursids where long-term population productivity is difficult or impossible to quantify.

Hair provides an excellent approach for examining physiological responses to food resource shortages as it can be chemically analyzed to determine both diet and steroid hormone levels. In contrast with serum and feces, which are commonly used for measuring steroid hormones in wildlife and reflect time periods of minutes to hours, hair reflects endocrine activity integrated over several months. Consequently, steroids in hair are insensitive to short-term stressors [7] and can be related to longer-term life history events and stages [8]. Steroid hormones are incorporated into growing hair via the blood vessel that feeds the hair follicle and/or from the follicle itself, which can synthesize steroids locally [8]–[10].

An increasing number of studies, most focusing on the glucocorticoid stress hormone, cortisol, have shown that steroid measurements from hair provide biologically meaningful information in humans, captive animals and wildlife [7], [11]–[18]. Recently, several studies have provided biological validation in ursids, including grizzly bears [17], [19], polar bears (*Ursus maritimus*) [16], [18], [20], [21], and Asiatic black bears (*Ursus thibetanus*) [22]. Notably, MacBeth [23] found relatively high levels of cortisol in hair from an emaciated grizzly and an emaciated black bear (*Ursus americanus*) compared with 151 other grizzly bears. Similarly, Malcolm et al. [22] documented higher cortisol in hair of Asiatic black bears kept under stressful conditions on a bile farm and those recently admitted to a shelter compared with bears already living at the shelter. Moreover, paired samples showed that cortisol in hair decreased as bears acclimatized to the shelter [22].

As a long-lived species that is acutely sensitive to large-scale anthropogenic disturbances [24], [25], grizzly bears serve as a model system for understanding the physiological effects of food resource declines. For millenia, grizzly bears (*Ursus arctos*) in coastal British Columbia, Canada, and beyond have evolved with abundant Pacific salmon (*Oncorhynchus* spp.) that becomes available each year during the autumn spawning event [26], [27]. Salmon allows bears to meet their energetic requirements more efficiently than a diet of plants alone [28]–[30]. Moreover, nutrients from salmon come at a critical time before hibernation when pre-denning fat reserves are positively correlated with over-winter survival and reproduction in the following year [31]–[33]. Among populations, grizzly bears with access to salmon have higher population density, body size and litter size [34].

Historically, salmon returns have been a relatively predictable annual event for coastal bears, though the number and timing of spawners varies among years and streams [35]. Despite some exceptions, there have been widespread or regional declines in salmon abundance through much of coastal British Columbia [36]–[38]. Today, fewer than 4% of streams monitored in coastal BC consistently meet their salmon escapement targets (i.e., number of salmon that escape human fishing nets and return to their natal streams to spawn) [39]. Notably, the hair of bears in North America grows for approximately six months from spring to fall [40]–[42], during which salmon are consumed for three months [Table 1].

**Table 1:** Approximate time line of hair growth and corresponding yearly natural history events of grizzly bears.

	May-October	November-April
Hair Growth	Hair grows, incorporating steroid hormones & isotopic dietary information [40]–[42]. **May**: old hair shed, new coat starts to grow.^1^	Hair stops growing over winter & does not incorporate steroid hormones or dietary information.
Bear Biology	**May-July**: Bears eat mainly vegetation & some terrestrial meat [61], [105]. Breeding season, increases in male-male aggressive interactions[79]. **Aug.-Oct**: Coastal bears aggregate on salmon streams. Bears gain body mass for over-winter survival & reproduction [31]–[33].	Hibernation. Cubs born. Bears may lose >30% of their body mass [31]

1 All hair samples collected in May were grown in the previous year.

To examine whether endocrine levels are potentially influenced by variation in salmon availability and consumption, we compared hormone levels in a population of coastal bears with access to salmon with an interior population without access to salmon. Among coastal bears only, we examined the relationship between hormones and salmon consumption (determined by stable isotope analysis). Between regions, we predicted that cortisol, as a general indicator of physiological stress, would be elevated in response to nutritional stress [43], [44] or social instability [45]. Among coastal bears, we predicted a negative relationship between cortisol and salmon consumption, reflecting either a nutritional or social benefit of access to more salmon.

To date, no studies have examined testosterone and progesterone in bear hair. Testosterone plays an important role in reproduction and also varies in relation to the social competitive environment above levels required for reproduction [46]–[49]. In particular, testosterone facilitates behavioral and physical traits necessary to win social conflicts in fitness-enhancing situations [49], [50]. Therefore, we predicted that testosterone would be elevated in coastal bears, where population density is higher and social interactions occur over temporally and spatially constrained salmon runs. Among coastal males, we predicted higher testosterone in males that consume more salmon, possibly reflecting a nutritional benefit of eating salmon or higher social density in areas where more salmon is available to be eaten.

Progesterone, which is elevated in females during pregnancy and pseudopregnancy, should be positively associated with population-level reproductive activity [51], because hair grows over the time interval that incorporates follicular development, ovulation, and mating. Given the higher productivity of bear populations with access to salmon [29], we predicted that progesterone would be higher in coastal compared with interior females.

## Materials and Methods

### Ethics statement

Samples were collected under animal care protocols approved by the Chancellor's Animal Research Committee at the University of California Santa Cruz (WILMc0904) and the Animal Care Committee at the University of Calgary (BI10R-01). Our sampling sites occurred in the traditional territory of the Heiltsuk Nation as well as in provincial parks. Permission to collect samples from these areas was granted by the Heiltsuk Integrated Resource Management Department and BC Parks (Park Use Permit Number 103586).

### Study areas and sample collection

**Figure 1:**
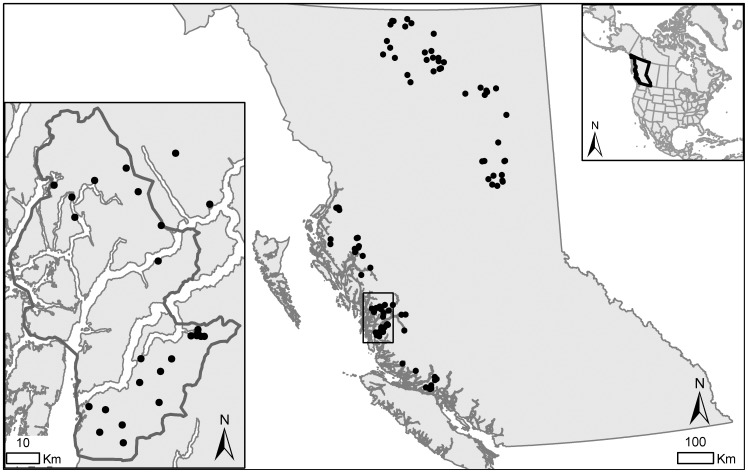
Bear hair collection areas. We collected grizzly bear hair samples (circles) from coastal and interior British Columbia (BC) in the springs of 2004–2011. The samples came either from government archives of hunted bears or hair snagging stations on the central coast of BC. The bottom left inset shows the coastal study area where we sampled from hair snagging stations in springs of 2009–2011.

We collected bear hair samples from coastal and interior BC (Fig. 1). On the coast, our core study area was located near Bella Bella (52°13′15.8”N, 127°45′28.4”W) where we collected hair samples using standard, grid-based DNA mark-recapture methods [52]–[54]. In a 2009 pilot year, we sampled over 2500 km^2^ at 92 barbed-wire hair-snagging stations placed within 5×5 km grid cells. In 2010 and 2011, we expanded the area to 5000 km^2^ with 71 snag stations in 7×7 km cells. We obtained additional hair from archived samples of grizzly bears killed in coastal BC in the springs of 2004–2009. These samples came from a larger area extending from Knight Inlet in the south (50°29′44.5”N, 131°36′30.5”W) to the Khutzeymateen (54°59′28.6”N, 122°36′13.8”W) grizzly bear management unit in the north [55]. Coastal bears assimilate a substantial portion of their yearly dietary protein from salmon [56]. These bears inhabit the coastal western hemlock biogeoclimatic zone of BC, which is characterized by high precipitation (average 2228 mm/year) and a temperature averaging 8°C [57].

For comparison with coastal bears, we obtained archived hair from bears in the interior of BC. The sampling extent ranged from the Moberly grizzly bear management unit in the south (55°32′25.4”N, 129°37′30.6”W) to the Hyland and Muskwa units in the north (59°46′8.6”N, 126°40′2.3”W). Bears in these regions eat plants and terrestrial meat and do not have access to anadromous salmon or kokanee [56]. In contrast with coastal bears, interior bears inhabit a region with lower precipitation (330–570 mm/year) and a continental climate characterized by warm summers, cool winters, and an average yearly temperature between -3 and 3°C [57], [58].

Regarding human interactions, both bear populations have coexisted with local First Nations for thousands of years; today, the human population density in both areas is relatively low compared with elsewhere in the province [58], [59]. Industrial activities such as logging occur in both areas but the extent of human activity, particularly road density, is higher in the interior [57], [60].

All hair samples were collected in spring and therefore reflected hair grown from spring to fall of the previous year (Table 1). Notably, bear hair grows—and incorporates hormones—at approximately one cm/month over the six month period when bears are most active [61]. We stored the samples in paper envelopes at room temperature in a dark, dry environment [28].

### Genetic Analyses

After collection, we sent all samples to a commercial laboratory (Wildlife Genetics International, Nelson, BC, Canada) where seven microsatellite markers were used to identify individual bears as well as their species and sex [62]. We used the remaining hair shafts for stable isotope analysis and hormone assays. When multiple samples collected from single or different snag stations were identified genetically as being from one individual in the same year, we pooled samples to obtain enough material for hormone assays.

### Stable isotope analysis to quantify salmon consumption

We prepared samples for stable isotope analysis as previously described [63], [64]. Subsequently, we sent the samples to the University of Saskatchewan's stable isotope facility where the ratios of nitrogen (^15^N/^14^N) and carbon (^13^C/^12^C) stable isotopes were measured using gas chromatography mass spectrometry. To estimate the proportion of salmon in the diet of each bear, we used a Bayesian mixing model [65], [66]. Following other studies of coastal grizzly bears, we assumed that bears' diets consist only of plant or salmon-based protein [56]. We used previously published estimates of anadromous salmon and plant stable isotope signatures, standard deviations, and fractionation rates [56].

### Analysis of steroids in hair

Our protocol for analyzing steroids in hair was similar to that previously published [67]. Additional details on hormonal assays and validations are provided as supporting information (Fig. S1, Table S1, Text S1).

### Bear density estimates

Across the province, we classified bear density in coastal and interior bears based on government estimates for each of the grizzly bear management units of BC [55]. In the grid-based coastal study area, we divided the study area into 10 units of 342–900 km^2^ each based on BC's conservation landscape units [68]. We estimated bear density within a landscape unit by dividing the number of genetically unique individuals detected in 2010 and 2011 by the number of hair snag stations (all sampled with the same effort) in that landscape unit. We used this approach to provide a generalized indicator of density based on bear detections at several nearby snag stations. Therefore, we used number of hair snags rather than unit area as our denominator in this calculation. Bear density estimates fit naturally into high and low classifications.

### Data Analysis

All analyses were conducted in R [69]. Before analysis, we removed outliers falling >2 SD from the mean for cortisol and/or testosterone (n = 4). Three of these samples had extreme values that were 15–37 times higher than the mean and were beyond the range of the hormone assays prior to dilution. The fourth sample was a multivariate outlier for cortisol and testosterone, identified using the gap test [70]. In future, and if similar outliers are a consistent finding, they may be an interesting subset to consider, possibly reflecting high physiological stress. However, they might also be caused by extreme cases of external contamination not removed by our wash procedure or an unidentified error in the laboratory. Here, we assumed the latter possibility and excluded these individuals from statistical analysis. One coastal bear was excluded for having a non-coastal dietary salmon signature; we suspected this was due to an error or mix-up in the database. To improve normality, we applied a negative reciprocal transformation to cortisol and testosterone and an arcsin transformation to our proportion of salmon in diet metric [71]. Progesterone did not require a transformation, possibly because of few samples from female bears (n = 21). We used t-tests to check for differences in samples collected from hunters and hair snags in coastal BC.

#### Comparison of coastal and interior bear populations

We developed candidate linear regression models to examine the effects of region, sex, and bear density on cortisol and testosterone. Model sets for both hormones included an interaction between region and sex because we predicted that males and females might respond differently to salmon availability across regions as well as a null model containing a constant. We ranked models using Akaike information criterion, corrected for small sample size (AICc), and considered our top model set to include all candidate models with a ΔAICc score <2. To assess the adequacy of top models, we plotted histograms of the residuals, residuals versus predictors and residuals versus predicted values. In addition, we examined Cook's distance as an indicator of influential observations. Variance inflation factors for top models ranged from 1.0 to 2.0 indicating low collinearity among variables. For comparisons of progesterone, we used a linear model to compare coastal (n = 15) to interior females (n = 9) and a t-test to examine differences between females (n = 21) and males (n = 4).

#### Salmon-hormone relationships in coastal male bears

For these analyses, we excluded female bears because of their small sample size (n = 15 of 70 bears). We first explored our prediction of a direct relationship between cortisol or testosterone and salmon consumption among all coastal bears (n = 55) using linear regression. In addition, we posited that the amount of salmon bears consumed in autumn would influence their nutritional and physiological state emerging from hibernation in the following year; therefore, we examined whether there was a lag between hormone levels and salmon consumption by examining trends over time. To address this question, we focused on cortisol and testosterone in coastal males from our grid-based study area (n = 28; where we had a field-based measure of relative density) to compare the effects of salmon consumption, year, and bear density using linear models, as described above. We centred and scaled the salmon consumption metric so that parameter estimates of variables would be comparable. Variance inflation factors for all models ranged from 1.0 to 1.4. Finally, we used f-tests and paired t-tests to examine trends in individuals detected (and measured with hormonal and isotopic assays) in two years of the study (n = 7).

## Results

### General patterns

Overall, the median cortisol concentration was 8.1 pg/mg [range: 5.3–26.1] in 113 hair samples, the median testosterone was 5.6 pg/mg [range: 3.1–21.1] in 112 samples, and the median progesterone was 26.2 pg/mg [range: 9.1–46.2] in 27 samples. Among coastal bears, cortisol and testosterone were similar in hair samples collected from hunters and snag stations so samples were pooled in subsequent analyses (cortisol: t = 0.27, df = 66, p = 0.79; testosterone: t = −0.23, df = 67, p = 0.82).

### Comparison of hormones in coastal and interior bear populations

**Figure 2:**
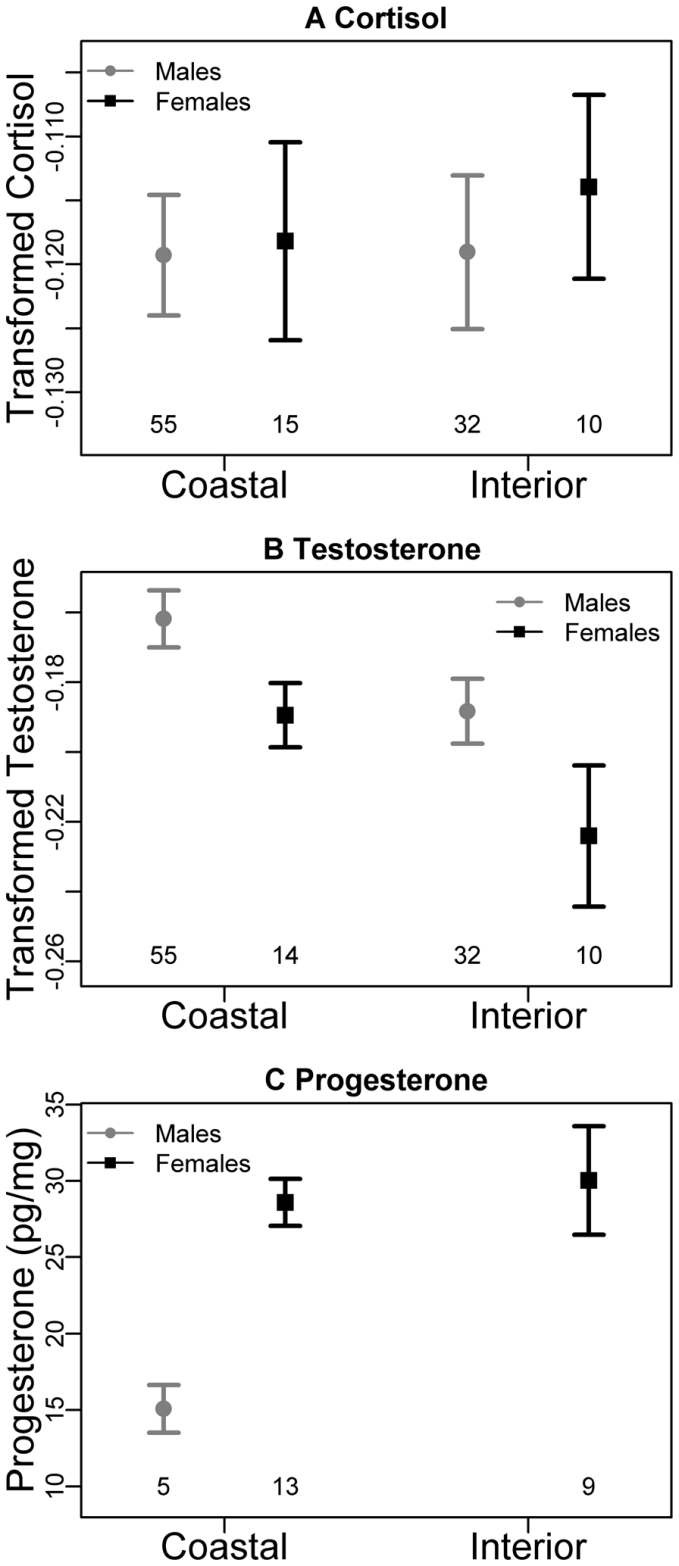
Mean and standard error for cortisol (A), testosterone (B), and progesterone (C) across regions and sexes. (A) Cortisol was similar between sexes and regions in grizzly bears. (B) Testosterone was higher in males and coastal bears. (C) Progesterone was higher in females. Cortisol and testosterone were reciprocal transformed (-1/x) to improve normality; progesterone is expressed in pg/mg of hair. Sample sizes are displayed below the error bars.

The most striking difference between regions was higher testosterone in coastal bears of both sexes, which is consistent with our prediction of higher social density among bears with access to salmon (Fig. 2B; Table 2). As expected, the top model also revealed higher testosterone in males than females (Fig. 2B; Table 2). By contrast, the top model for cortisol was the null model, suggesting no differences between coastal and interior bear populations or sexes (Fig. 2A; Table 2). Notably, bear density was not an important predictor of cortisol or testosterone (Table 2). Progesterone did not differ between regions (Fig. 2C; Table 2) and was higher in females (t = −6.2, df = 15, p<0.001; Fig. 2C).

**Table 2:** Estimates and standard error (in parentheses) of parameters in the top models (ΔAICc≤2) describing cortisol, testosterone and progesterone in hair of coastal (n = 70) and interior (n = 42) grizzly bears.

Response (models compared)	Intercept	Sex = Female	Region = Interior	Sex* Region	Bear Density^a^
Cortisol (10)	−0.119* (0.003)	–	–	–	–
Testosterone (8)	−0.161* (0.007)	−0.031* (0.013)	−0.028* (0.011)	–	–
Progesterone^b^ (2)	29.2* (1.67)	S^c^	–	NE^d^	NE^d^

* Significant at α = 0.05

a Based on government estimates of density in grizzly bear management units

b Females only

c Significant, a separate t-test showed that females (n = 21) had higher progesterone than males (n = 4)

d Not examined

### Salmon-hormone relationships in coastal male bears

**Figure 3:**
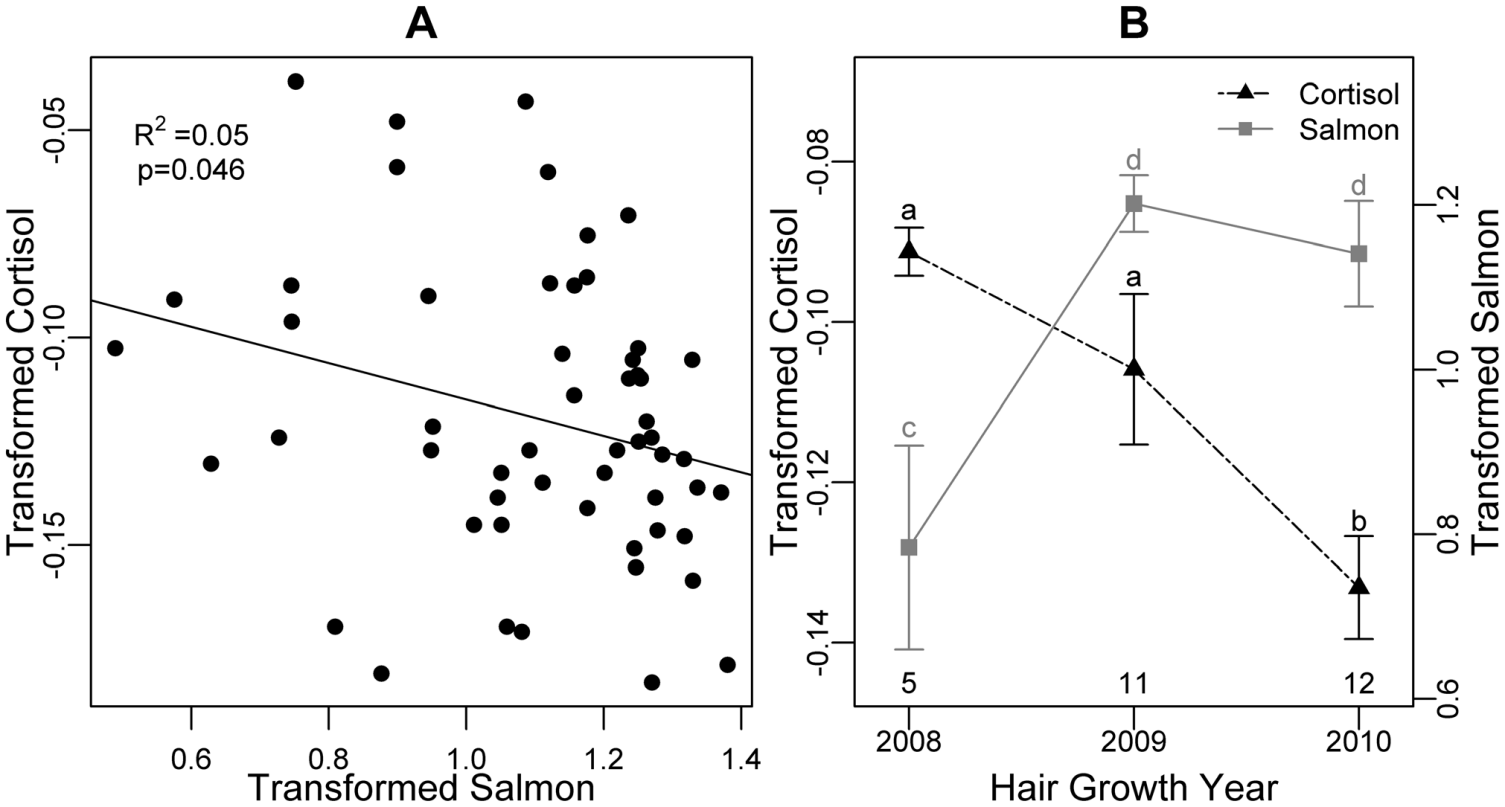
Evidence suggesting a relationship between cortisol and dietary salmon in male coastal grizzly bears. (A) Across all coastal males (n = 55), cortisol was weakly but negatively correlated with dietary salmon. (B) In the smaller, grid-based study area, mean cortisol was lower in 2010 following a year of high population-level dietary salmon than in 2009 following a year when bears ate less salmon. Note that we have no dietary salmon data from 2007, which might influence cortisol in 2008. (B). Letters above the error bars show significantly different groups. Error bars represent standard error. Sample sizes are presented below the error bars. To improve normality of residuals, cortisol was reciprocal-transformed (-1/x) and dietary salmon was arcsin transformed.

As predicted among all coastal males (n = 55), hair cortisol decreased with increasing dietary salmon, though very marginally (adj R^2^ = 0.06, F_1,53_ = 4.2, p = 0.046; Fig. 3A). In the smaller coastal study area that we sampled consistently in three years (n = 28), our model selection approach identified the effects of salmon consumption and year as being important predictors of cortisol (Table 3). Similar to the trend in the larger dataset, cortisol decreased marginally with increasing salmon consumption. Moreover, the top model set revealed that cortisol was higher in 2008 and 2009 after years of low average salmon consumption compared with 2010 after a year of higher salmon consumption (Fig. 3B; Table 3).

**Table 3:** Estimates and standard error (in parentheses) of parameters in the top models (ΔAICc≤2) describing relationships between hormones and variables relating to salmon consumption among coastal male bears (n = 28).

Response (models compared)	ΔAICc	Intercept	Salmon Consumption	Year^a^ (2008)	Year^a^ (2009)	Bear Density^b^ ( = High)
Cortisol (8)	0	−0.131 (0.007)	−0.011 (0.006)	0.026 (0.015)	0.030* (0.010)	–
	0.86	−0.133 (0.007)	–	0.042* (0.013)	0.027* (0.013)	–
Testosterone (8)	0	−0.161 (0.011)	–	–	–	–
	0.76	−0.125 (0.023)	−0.022 (0.012)	–	–	−0.049 (0.028)
	1.25	−0.161 (0.011)	−0.012 (0.011)	–	–	–
	1.44	−0.142 (0.022)	–	–	–	−0.025 (0.025)

* Significant at α = 0.05

a 2010 is the reference category

b Based on field estimates of bear density; low bear density is the reference category.

In contrast with our prediction, there was no evidence of a relationship between testosterone and dietary salmon among all coastal males (adj R^2^ = 0, F_1,53_ = 0.97, p = 0.33). In the grid-based coastal study area, the top model for testosterone was the null, revealing that the variables we examined explained little of the variability in hair testosterone. However, the top model set included weak effects of bear density and salmon consumption (Table 3). In contrast with our predictions, testosterone decreased with increasing salmon consumption and was lower in areas of high bear density.

**Figure 4:**
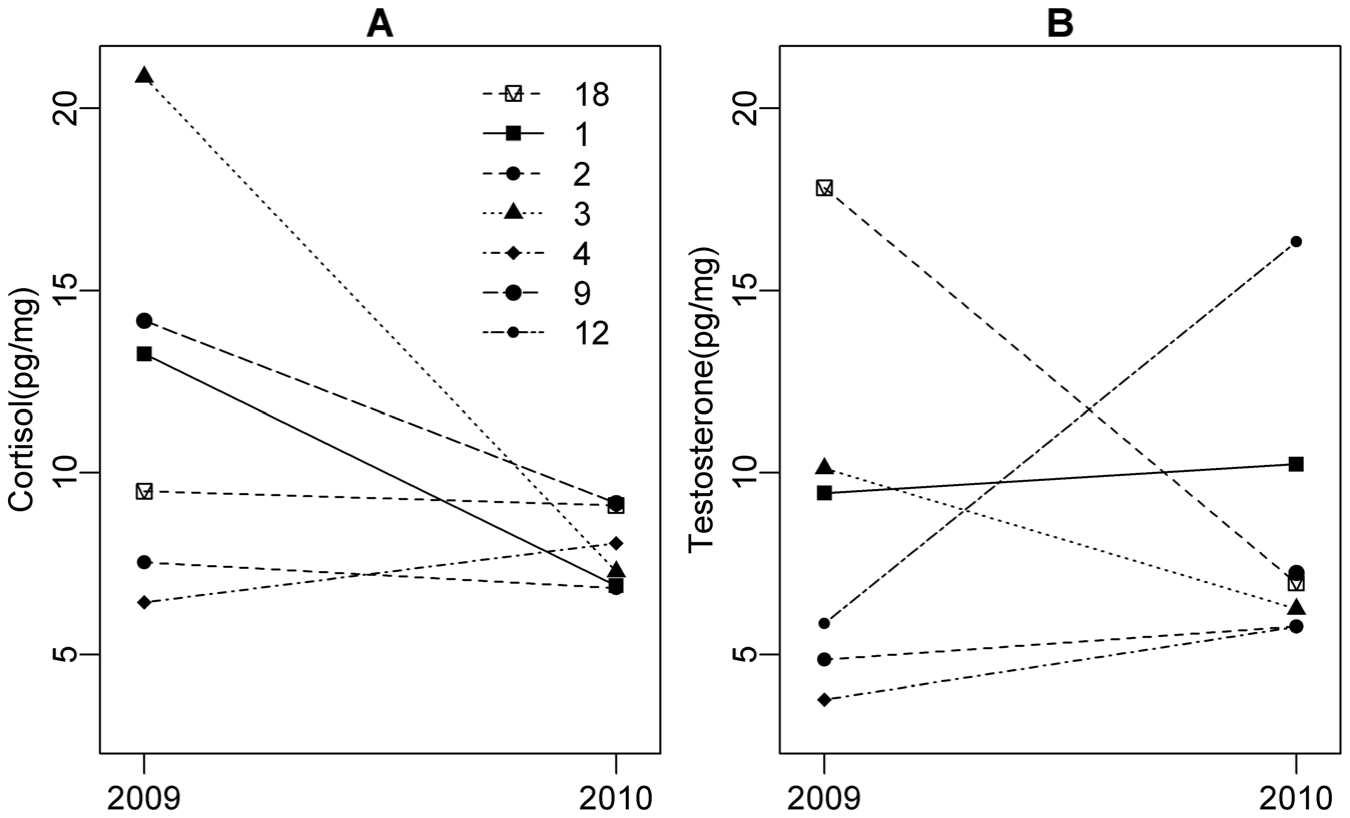
Hormones in male bears detected in 2009 and 2010 reflected trends at the population level. Cortisol was generally higher and more variable in 2009 following a year of low salmon abundance compared with 2010 after a year of relatively high salmon abundance (A). Testosterone did not show a consistent trend between years (B). Each line type and point symbol represents an individual bear. No data were included from 2008 because none of the samples from recaptured bears in that year had sufficient material for hormonal analysis.

Trends in the seven bears sampled in both years reflected those at the population level; these bears had more variable cortisol in 2009 than in 2010 (F_5,5_ = 25.3, p = 0.003; Fig. 4A). Cortisol levels in several bears were lower in 2010 than 2009, but the difference was not significant (paired t = 1.80, df = 5, p = 0.13). Testosterone did not show a consistent trend between years (t = 0.03, df = 5, p = 0.98; Fig. 4B).

## Discussion

### General patterns

Hormone measurements in hair—which reflect long-term endocrine information—provide novel insights into the physiological responses of wildlife to environmental change [8], [16]–[18], [20], [21]. Interestingly, the cortisol values in our study (median: 8.1 pg/mg, range: 5.3–26.1) were higher than those previously reported in 151 live-captured grizzly bears from Alberta, Canada (median: 2.8 pg/mg, range: 0.6–43.3 pg/mg; [17]). These differences are probably methodological but could also relate to differences in population densities, habitat, genetics, or the dynamics of natural and anthropogenic stressors.

Our findings show that immunoreactive progesterone and testosterone can be measured relatively easily in addition to cortisol from the same hair sample. Moreover, hormone concentrations revealed expected differences between sexes. Elevated testosterone in hair of males reflects higher testosterone levels in males during the breeding season, which occurs in June and July [72]. Similarly, progesterone is elevated in female bears following fertilization, which occurs in spring or early summer [73]. Though the corpora lutea are mostly dormant until implantation occurs in late fall, they produce enough progesterone that levels are elevated above baseline in pregnant and pseudo-pregnant females [74], [75].

### Comparison of coastal and interior bear populations

This study also provides insight into the physiological implications of living in an environment with a nutritious but seasonally and spatially constrained resource. Salmon provides nutritional benefits to bears [31], [32], [34]; however, higher testosterone in coastal bears could have fitness costs such as increased energetic expenditure and risk of injury from intraspecific interactions, as well as impaired immunity [47], [48]. Differences in testosterone between coastal and interior bears could also relate directly to diet. Spawning salmon have high levels of androgens, which could potentially increase circulating testosterone levels in bears, which have access to salmon for approximately three of the six months of hair growth [76]. However, we found no support for this possibility as dietary salmon and testosterone were not correlated in coastal males. Moreover, cortisol was not higher in coastal bears, even though spawning salmon have extremely high levels of glucocorticoids [77].

The higher testosterone levels of coastal bears might relate to their larger body size compared with interior bears. Previous studies of bears have shown that testosterone is positively linked with body size in males during the breeding season [78], [79]; however, it is not clear whether the same trend would occur between populations differing in average size or whether the relationship would be detectable in hair, which integrates endocrine activity during the breeding and non-breeding periods.

Our findings show that testosterone did not vary with provincial estimates of bear density. It is possible that the spatiotemporal distribution of resources at scales smaller than region is a more important mediator of social interactions than the number of bears in a region [80], [81]. Indeed, several characteristics of habitat and resource availability would affect the frequency and type of social interactions (i.e., social density) between regions. Whereas interior bears use a variety of habitats from the treeline to the alpine [58], [82], coastal bears spend most of their time along valley bottoms due to less usable habitat on the coast [83]. Food sources also differ; interior bears feed on vegetation and opportunistically on ungulates [58], [80], [84]. These bears use productive habitats such as burns and berry patches but the feeding aggregations are less pronounced compared with those on spawning salmon streams [85], [86]. Indeed, well-described social interactions over access to salmon often lead to aggressive encounters and the establishment of dominance hierarchies [80], [86]. An influx of bears from the interior to salmon spawning streams in the fall would make the social dynamics particularly intense.

In coastal female bears, higher testosterone compared with interior females might reflect higher reproductive rates. However, this is unlikely the only explanation as the latter stages of pregnancy—when testosterone levels are highest—occur in winter when hair is not growing and does not incorporate steroid hormones. Alternatively, higher testosterone in coastal females might be modulated by social conditions. Though usually considered with respect to reproductive traits in males, testosterone in females has been linked with defending resources and acquiring food [87], [88]. In female bears, elevated testosterone might be advantageous in obtaining salmon, a food that increases reproductive success but that can be difficult to obtain because of intense competition with other bears [34], [86]. Testosterone in female bears could also relate to aggressive encounters to prevent infanticide by males or other females on salmon spawning streams [89], [90]. Additional studies of female bears with and without cubs would be helpful in understanding factors affecting testosterone levels in females.

In contrast with testosterone, cortisol was similar between regions, suggesting that coastal and interior bears experience similar levels of physiological stress or have different baseline cortisol levels. One possible explanation for the lack of a difference is that the nutritional benefit of access to salmon is overwhelmed by costs imposed by higher social density among coastal bears. Similarly, there was no evidence that progesterone differed between populations, possibly because we were not able to account for age, reproductive history (e.g., inter-birth interval and presence of cubs) or reproductive success (i.e., successful versus pseudo-pregnancies).

### Relationships between hormones and salmon consumption among coastal bears

Two lines of evidence support our prediction that cortisol would be higher in coastal bears that eat less salmon. Among coastal males, there was a weak but significant negative relationship between cortisol and dietary salmon. Elevated cortisol could be an adaptive response to food shortage to mobilize fat [73]. Cortisol might also play a role in bone resorption during periods of nutritional stress [91] or affect the amount and type of foods consumed [92]. Moderately elevated glucocorticoid levels could also improve foraging efficiency during reduced food availability by enhancing spatial memory [93], increasing exploratory behavior [94], or promoting innovation of novel foraging approaches [95].

The negative association between diet and cortisol could also reflect lower social tension when there are more salmon to eat. Grizzly bears in coastal areas have a social hierarchy with larger, older males being dominant over smaller, younger males [86]. Bears would be more tolerant of each other and would not have to vie for access to salmon when they are abundant [96].

Additional evidence of a direct relationship between cortisol and dietary salmon comes from our grid-based study area where we sampled consistently over three years. Cortisol was higher in 2008 and 2009 after years of low dietary salmon than in 2010 after a year of higher dietary salmon. This suggests that the amount of salmon bears consume in fall influences circulating cortisol and therefore deposition in hair in the following spring. Previous studies have established that bears entering hibernation in poor body condition have lower body mass and reproductive success in the following year [31]–[33]. Elevated cortisol in spring might play a role in minimizing further weight loss after a year of low dietary salmon by maximizing energy intake from low-fat, herbaceous foods, which are available in spring [97], [98].

More data over several years, especially on individuals sampled multiple times, would help determine whether cortisol levels in hair relate to dietary salmon in the year of hair growth or during the spawning salmon season in the previous year. It would also be possible to segment hair corresponding to spring and fall periods in order to partition whether cortisol relates to the previous or the same-year dietary salmon [99], [100]. In future studies, it will be important to monitor factors such as temperature, productivity of herbaceous foods, and precipitation, which could affect hormone levels and vary among years [101]. More studies are required to determine whether elevated cortisol has negative fitness consequences for bears, as has been shown for corticosteroids deposited into hair of polar bears [16] and feathers of sparrows [102].

Contrary to our prediction, we found only weak evidence of a relationship between testosterone and salmon consumption and the trend was opposite to our initial prediction. The negative association between testosterone and salmon consumption might occur if there is less competition for salmon when more salmon is available. This possibility could be further explored by examining the effect of salmon availability, which would influence social conditions, in addition to salmon consumption.

## Conclusion

This work shows that variation in salmon abundance and consumption affects bears by altering nutritional and/or socially-mediated physiology. If salmon returns consistently decline in the future, grizzly bears that do not obtain enough salmon might experience chronically elevated cortisol and testosterone via increased nutritional and/or social stress, with unknown, but probably adverse, fitness costs. Moreover, our findings underscore the importance of considering implications for wildlife that share resources with humans as part of ecosystem-based fisheries management strategies [103], [104]. Ultimately, this work adds to a growing understanding of the value of measuring stress and reproductive measures in wildlife hair as indicators of broader population health and processes [8], [16], [19].

## Supporting Information

Figure S1 **Parallelism of assay standards and diluted hair extracts for (A) cortisol, (B) testosterone, and (C) progesterone**. Hair concentrations were measured in pg/mg of hair; regression lines were shifted on the x-axis for better visualization.
(TIF)

Table S1 **Validations for analysis of bear hair using commercial cortisol, testosterone and progesterone enzyme immunoassays (Salimetrics, Philadelphia, Pennsylvania, USA).**
(DOC)

Text S1 Supplementary info
(DOC)
