# Supplementary material for: Stress and Reproductive Hormones in Grizzly Bears Reflect Nutritional Benefits and Social Consequences of a Salmon Foraging Niche
Source: PLoS One. 2013 Nov 27;8(11):e80537. doi: 10.1371/journal.pone.0080537 (PMC3842319; doi:10.1371/journal.pone.0080537)
Supplement: Table S1 — Validations for analysis of bear hair using commercial cortisol, testosterone and progesterone enzyme immunoassays (Salimetrics, Philadelphia, Pennsylvania, USA). (DOC) [file pone.0080537.s002.doc]

**Table S1. Validations for analysis of bear hair using commercial cortisol, testosterone and progesterone enzyme immunoassays (Salimetrics, Philadelphia, Pennsylvania, USA)**.

|  | **Cortisol** | **Testosterone** | **Progesterone** |
| --- | --- | --- | --- |
| Linearity^1^ (p-value) | 0.030 | 0.799 | 0.731 |
| Sensitivity^2^ (pg/mL) | 30 | 1 | 5 |
| Recovery^3^ (%) | 95.5 | 93.4 | 102.5 |
| Hair required (mg) | 20 | 1 | 2 |
| Number of plates/runs | 15 | 15 | 5 |
| Intra-assay CV^4^ (%) |  |  |  |
| Hair quality control | 4.9 | 5.3 | 4.2 |
| Kit high quality control | 3.4 | 3.8 | 7.4 |
| Kit low quality control | 8.2 | 7.7 | 13.3 |
| Hair powder repeats (n=6) | 4.4 | 6.3 | 5.6 |
| Hair extract repeats (n=6) | 7.9 | 6.6 | 6.2 |
| Inter-assay CV^4^ (%) |  |  |  |
| Hair quality control | 6.2 | 10.3 | 6.4 |
| Kit high quality control | 7.6 | 7.5 | 8.6 |
| Kit low quality control | 5.8 | 13.9 | 25.1 |

^1^Linearity was assessed by analysis of covariance

^2^Sensitivity data were provided with the kit documentation

^3^Recovery was assessed by comparing three samples spiked before and three spiked after extraction

^4^Intra- and interassay coefficients of variation (CV) were assessed using quality controls provided with the kit and hair extracts run in duplicate twice per plate.
