# Supplementary material for: Stress and Reproductive Hormones in Grizzly Bears Reflect Nutritional Benefits and Social Consequences of a Salmon Foraging Niche
Source: PLoS One. 2013 Nov 27;8(11):e80537. doi: 10.1371/journal.pone.0080537 (PMC3842319; doi:10.1371/journal.pone.0080537)
Supplement: Text S1 — Supplementary info (DOC) [file pone.0080537.s003.doc]

***Extraction of steroids from hair***

Our protocol for extracting steroids from hair was similar to that described in [1]. Starting with 50 mg of hair, we performed two washes with 40 mL of water followed by two washes with 40 mL of isopropanol on a plate rotating at 130 rpm for three minutes per wash. After each set of washes, we thoroughly dried the samples and powdered them in a ball mill (Mixer Mill 200, Retsch, Haan, Germany). We weighed 30 mg of powdered hair into a glass scintillation vial and added HPLC grade methanol (Omnisolv; VWR, Mississauga, Ontario, Canada) at a concentration of 100 µL/mg of hair. Next, we sonicated the samples for 30 minutes followed by an 18 hour extraction at 50˚C on a plate rotating at 160 rpm. After extraction, we centrifuged the samples for 30 minutes at 4000 rpm, aliquotted the supernantant into separate tubes for testosterone (100 µL), progesterone (200 µL) and cortisol (2000 µL) assays, and evaporated the methanol under a gentle stream of nitrogen. For samples where less than 30 mg of hair was available, we aliquotted smaller volumes of the extract, corresponding to 5, 10, or 15 mg of hair. Prior to analysis, we reconstituted samples in 6.5 µL of methanol followed by 123.5 µL of assay diluent. We measured immunoreactivity to cortisol, progesterone, and testosterone using commercial kits designed for use on saliva (Salimetrics, Philadelphia, Pennsylvania, USA). To minimize inter-assay bias, we randomly assigned samples to plates and where possible, analyzed samples from the same bear detected in multiple years on one plate.

***Immunoassay Validation Procedure***

We assessed parallelism for each assay by comparing a serially diluted extract of hair from each species with assay standards. We used analysis of covariance to compare the slopes of the regression lines for standards and serially diluted hair samples. We assessed repeatability in several ways. To test variability among hair samples due to the entire procedure from weighing to measuring immunoreactivity, we prepared six separate samples from the same pool of hair and assayed each sample in duplicate on one plate. At the same time, we extracted hormones from a large sample of the pooled hair and measured sub-samples of the extract in duplicate, six times; this would provide an indication of variability related to the reconstitution and quantification steps. To quantify the intra- and inter-assay coefficients of variation, we ran high and low quality controls provided with the commercial assay on every plate. As an additional control, we measured an aliquot from a pool of hair twice per plate. We assessed extraction efficiency by adding a known amount of steroid to three hair samples prior to extraction and comparing the average result to that from three hair samples spiked after the extraction.

We reported the cross-reactivity for the cortisol assay in [1]. According to the kit insert, the cross-reactivity for testosterone was: aldosterone (<0.004%), androstenedione (1.157%), corticosterone (<0.004%), cortisol (<0.004%), cortisone (<0.004%), 11-deoxycortisol (<0.004%), 21-deoxycortisol (0.004%), DHEA (<0.004%), dianabol (0.489%), dihydrotestosterone (36.4%), epitestosterone (0.165%), 11-hydroxytestosterone (1.90%), 19-nortestosterone (21.02%), epitestosterone (0.165%), estradiol (0.025%), estriol (0.012%), estrone (0.005%), progesterone (0.005%), 17 α-hydroxyprogesterone (<0.004%), and transferrin (<0.004%). The cross-reactivity reported for the progesterone kit was: prednisolone (0.0021%), prednisone (0.0038%), cortisone (0.0106%), 11-deoxycortisol (0.0195%), 21-deoxycortisol (0.0082%), 17 α-hydroxyprogesterone (0.0723%), dexamethasone (0.0014%), triamincinolone (<0.004%), corticosterone (0.1924), testosterone (<0.004%), DHEA (<0.004%), cortisol (<0.004%), transferrin (<0.004%), aldosterone (<0.004%), estradiol (<0.004%), estrone (<0.004%), and estriol (<0.004%).

***Results from Hormone Assay Validations***

We were able to detect and quantify three steroid hormones in grizzly bear hair using a simple extraction followed by commercial immunoassays. Our procedure was similar to those previously published for analyzing cortisol and androgens in hair of captive and free-ranging wildlife [2-4]. Results from validations of each assay are summarized in Table S1. Based on analysis of different amounts of hair, 20 mg was optimal for cortisol, 1 mg for testosterone and 2 mg for progesterone. Using these amounts of hair, all samples we analyzed were above the detection limit of the three assays. Two samples initially fell above the range of cortisol or testosterone assays and were diluted and re-run.

Visual inspection showed reasonable parallelism of assay standards and diluted hair extract, though cortisol showed moderate departure from parallelism (Fig. S1). An analysis of covariance confirmed that the slope of the line for the cortisol hair extract was not parallel with that of serially diluted standards (p=0.03, Table S1). This suggests that there are compounds in grizzly bear hair that cross-react with the cortisol immunoassay in a non-linear fashion. Though not ideal, we considered the degree of departure from parallelism tolerable [5]. Parallelism tests for testosterone and progesterone were non-significant (Table S1).

Recovery was acceptable for the three assays. The intra- and inter-assay coefficients of variation were satisfactory (<15%) with the exception of the low control for progesterone which had an inter-assay CV of 26.6%. No samples were in this range so poor repeatability in the lower portion of the curve would have had little influence on the overall results.

**Supplementary References**

1. Bryan HM, Adams AG, Invik RM, Wynne-Edwards KE, Smits JE (2013) Hair is a more repeatable reflection of baseline cortisol than saliva or feces in dogs. J Am Assoc Lab Anim Sci 52: 189-196.

2. Koren L, Mokady O, Karaskov T, Klein J, Koren G, et al. (2002) A novel method using hair for determining hormonal levels in wildlife. Anim Behav 63: 403-406.

3. Davenport MD, Tiefenbacher S, Lutz CK, Novak MA, Meyer JS (2006) Analysis of endogenous cortisol concentrations in the hair of rhesus macaques. Gen Comp Endocrinol 147: 255-261.

4. Macbeth BJ, Cattet MRL, Stenhouse GB, Gibeau ML, Janz DM (2010) Hair cortisol concentration as a noninvasive measure of long-term stress in free-ranging grizzly bears (*Ursus arctos*): considerations with implications for other wildlife. Can J Zool 88: 935-949.

5. Lee JW, Devanarayan V, Barrett YC, Weiner R, Allinson J, et al. (2006) Fit-for-purpose method development and validation for successful biomarker measurement. Pharm Res 23: 312-328.
